# A rare case of multiple giant colonic diverticula successfully treated with laparoscopic sigmoidectomy

**DOI:** 10.1093/jscr/rjab475

**Published:** 2021-10-21

**Authors:** Clay M Merritt, Chuheng Xing, Mary R Schwartz, Harold R Bailey, Jeffrey L Van Eps

**Affiliations:** Department of Surgery, Colon and Rectal Surgery, Fort Belvoir Community Hospital, Fort Belvoir, VA, USA; Department of Surgery, Section of Colon and Rectal Surgery, UTHealth Science Center at McGovern Medical School, Houston, TX, USA; Department of Pathology and Genomic Medicine, Houston Methodist Hospital, Houston, TX, USA; Department of Surgery, Section of Colon and Rectal Surgery, UTHealth Science Center at McGovern Medical School, Houston, TX, USA; Department of Surgery, Section of Colon and Rectal Surgery, UTHealth Science Center at McGovern Medical School, Houston, TX, USA

**Keywords:** colonic diverticulosis, giant colonic diverticulum, minimally-invasive surgery, colectomy

## Abstract

Colonic diverticulosis is pervasive in Western society, with over half of individuals over the age of 60 carrying the diagnosis. A Giant Colonic Diverticulum (GCD) is a rare presentation of diverticulosis, involving one or more colonic diverticula that measure 4 cm or greater. Less than 200 reports of GCD have been published in the literature. Almost all GCD patients present with symptoms, with abdominal pain being the most common. Diagnosis is usually made with CT imaging and recommended treatment is segmental colectomy. We present an atypical case of GCD with an asymptomatic presentation, initial diagnosis made during endoscopy and a minimally invasive resection of multiple GCD within the same patient.

## INTRODUCTION

Colonic diverticulosis is pervasive in the Western world. Nearly 80% of individuals over the age of 80 have diverticulosis of the colon [1]. Diverticula form at anatomic ‘weak’ points where arteries enter the circular smooth muscle of the colon wall, allowing herniation of mucosal and submucosal layers. In contrast to ‘true’ diverticula, which involve all layers of the bowel wall and are most often congenital, these outpouchings signify ‘false’ or ‘pseudo’ diverticula [2]. Most are small, measuring less than a few centimeters (cm) in greatest dimension, but a diverticulum measuring 4 cm or greater is defined as a giant colonic diverticulum (GCD), with reports of GCD over 20 cm. In a review of the literature, Nigri *et al.* [3] found less than 200 reported cases of GCD. The pathogenesis of GCD is largely unknown but the ball-valve theory is most common, hypothesizing that chronic high pressure from gas entering the diverticulum but unable to exit due to occlusive debris or inflammation at the diverticular neck stretches the tissues over time. The rising pressure is relieved either via diverticular decompression intraluminally or free perforation intraperitoneally [4–6].

McNutt *et al.* [5] defined three types of GCD (Table 1). Type one is the result of a normal, smaller, pseudo-diverticulum slowly increasing in size with intact mucosa in the diverticular wall. Type two describes a pseudo-diverticulum that has perforated and formed a chronic abscess cavity, as might occur after treatment of diverticulitis by laparoscopic washout without resection [7]. Type three is a true, congenital, diverticulum with all layers of the colon wall present. Most patients are symptomatic (93%), with abdominal pain the most common (69%) presenting symptom [3]. Diagnosis is usually made with imaging and computed tomography (CT) scan is the study of choice [8]. Endoscopy can also serve as a means of diagnosis, but increased risk of perforation limits its use. Although diverticulectomy has been reported, most recommend surgical treatment with segmental colectomy [3]. Many authors have reported successful laparoscopic resections for GCD; however, the majority of resections reported to date have been performed open—likely reflecting the common high acuity presentation that may include peritonitis or perforation [3, 9, 10]. We present an atypical case of multiple GCD with an asymptomatic presentation and an endoscopic mode of diagnosis, treated by successful minimally invasive segmental resection.

## CASE REPORT

**Figure 1:**
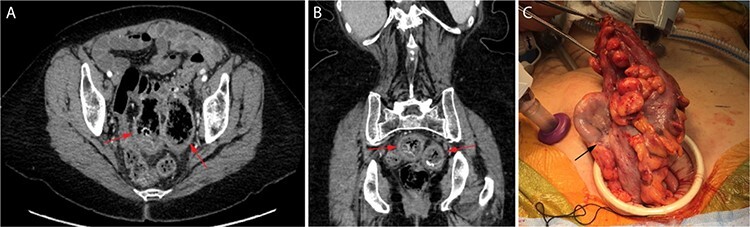
Cross-sectional CT imaging readily identified the two large GCD (red arrows) on both axial (A) and coronal (B) view. View of the largest GCD (black arrow) during specimen extraction intra-operatively (C).

A 68-year-old woman with history of hypertension, breast cancer, gastroesophageal reflux disease, osteoarthritis, hypothyroidism and prior GI bleeding with frequent NSAID use underwent diagnostic colonoscopy to evaluate anemia to 9.6-g/dL refractory to iron supplementation. Prior abdominal surgery included umbilical hernia repair and caesarian section. She denied abdominal symptoms including pain. Colonoscopy identified widespread left-sided diverticulosis and two large, cavernous areas partially filled with dark debris in the sigmoid colon without active bleeding. CT imaging confirmed severe diverticulosis with two GCD arising from the posterior wall of the proximal and mid-sigmoid colon measuring 6.8 × 4.4 × 4.3 cm and 6.1 × 5.2 × 3.9 cm, respectively (Fig. 1A and B). Mild wall thickening and trace, low-attenuation fluid in the adjacent sigmoid mesentery were suggestive of an inflammatory state without evidence of perforation. The patient was appropriately counseled and offered laparoscopic sigmoidectomy.

**Table 1:** Types of GCD

Type	Description
Type I	A normal smaller diverticulum that increases in size without perforation and maintains some mucosa in the diverticulum wall
Type II	Diverticulum results from perforation and formation of an abscess with a diverticulum wall consisting of fibrotic granulation tissue
Type III	Diverticulum contains all layers of the colon wall: serosa, muscularis, submucosa and mucosa

Intra-operatively, the two GCD were seen extending well into the pelvic cavity with minimal scarring and no evidence of perforation. Sigmoid colectomy was performed to an area of pliable, descending colon and specimen extraction was performed via a Pfannenstiel incision (Fig. 1C) before performing an end-to-end, circular stapled coloproctostomy anastomosis. The patient recovered well, with return of bowel function on post-operative day 4 and discharge home on post-operative day 6. Her anemia corrected with ongoing iron supplementation, and she experienced no complications within 10 months of follow-up.

**Figure 2:**
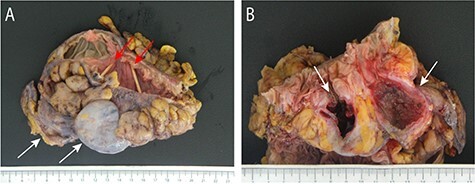
The resected segment of sigmoid colon and proximal rectum demonstrates the two known GCD (white arrows) with notable absence of external exudate and their luminal ostia (red arrows) marked by toothpicks (**A**). Open cross-section of the specimen demonstrates markedly narrow diverticular ostia (white arrows) with smaller adjacent (left) diverticular openings (**B**). The diverticulum on the right shows granular, polypoid mucosa with focal ulceration, while the GCD on the left has extensive ulceration and a thickened, fibrotic wall without perforation.

**Figure 3:**
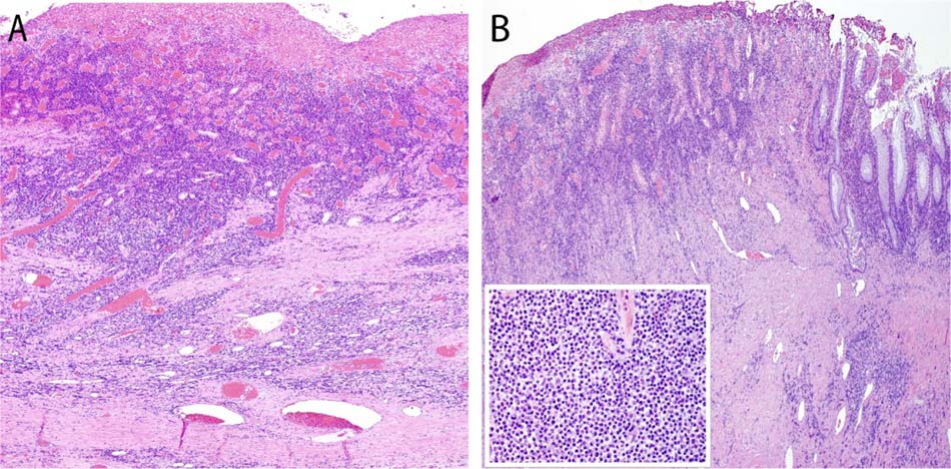
Photomicrographs of one GCD showing extensive ulceration (**A**) with underlying fibrosis (B) and marked submucosal plasma cell infiltration (inset, H and E, original magnification ×40; inset original magnification ×400). There is a small portion of non-ulcerated mucosa at far right for reference (**B**).

Macroscopic pathologic examination confirmed two giant diverticula with narrow ostia connecting them to the colonic lumen adjacent to more typical, smaller diverticula (Fig. 2). Microscopic examination confirmed the GCD to be ‘false’ or ‘pseudodiverticula’ with only mucosa and submucosa present in the diverticular wall (Fig. 3). Significant fibrosis and marked submucosal plasma cell infiltration were present, features of a chronic inflammatory state without perforation (Fig. 3), consistent with a type I GCD.

## DISCUSSION

Less than 200 cases of GCD have been reported since the first recorded report in 1946, making it a rare variant of an exceedingly common condition [3, 11]. Most patients are symptomatic at presentation, primarily reporting abdominal pain [3]. Despite having two GCD, our patient was asymptomatic, placing her in the overwhelming minority of GCD patients. Some authors have noted endoscopy to be ineffective in diagnosing GCD with limited visibility from narrow ostia or unsafe due to increased perforation risk [12]. Our patient, similar to a case described by Mehta *et al.*, safely underwent colonoscopy to rule out malignancy as the cause of her anemia and was discovered to have two GCD [12]. Notably, the patient had history of frequent NSAID use which has been linked to nearly a threefold increased risk of diverticular bleeding [13].

A review by Steenvoorde *et al.* [6] found that only 9% of patients present with multiple GCD, which can make surgical approaches more complex and highlights the importance of CT imaging for surgical planning. To the best of our knowledge, this is the first reported laparoscopic colectomy involving multiple GCD. Of the three types, this patient’s GCD is compatible with type 1 as mucosa was present in the walls of the non-perforated diverticula (Fig. 2). The inherent perforation present in patients with type II GCD may prove more technically challenging and has historically mandated an open surgical approach, especially for fulminant diverticulitis. Very large GCD over 10 cm may be difficult to resect using a minimally invasive approach given concerns of iatrogenic injury with specimen handling and limitations for extraction of such a large specimen. Ultimately, surgical approach must be tailored to each individual patient’s clinical presentation and unique anatomy. Our technique did not differ from the standard laparoscopic sigmoidectomy technique. This case confirms that GCD may be asymptomatic and demonstrates the feasibility and safety of minimally invasive surgical techniques for patients with multiple GCD.

## FUNDING

None.
